# Physiotherapy Rehabilitation to Recuperate a Patient From an Intertrochanteric Fracture: A Case Report

**DOI:** 10.7759/cureus.27660

**Published:** 2022-08-03

**Authors:** Shivani S Lalwani, Deepak S Jain, Pratik A Phansopkar, Tasneem M Lakkadsha, Sakina S Saifee

**Affiliations:** 1 Department of Physiotherapy, Ravi Nair Physiotherapy College, Datta Meghe Institute of Medical Science, Wardha, IND

**Keywords:** comprehensive rehabilitation, post-fracture surgery, harris hip score, quality of life, intertrochanteric fracture rehabilitation, physiotherapy treatment, proximal femur fracture, case report, dynamic hip-screw fixation

## Abstract

Intertrochanteric fracture is a prevalent condition among older adults, and it is becoming more so as the population is aging. A 52-year-old man was reported to the hospital with symptoms of pain and swelling in the right hip since the morning. The patient reported a history of unexpected slips and falls in the morning. An X-ray was taken of both hips, and an intertrochanteric fracture was identified. After one month post-fracture, a dynamic hip screw (DHS) was used to perform open reduction internal fixation (ORIF). Early mobility, appropriate lower limb strength, pain reduction, and quality of life are all significant determinants. As evidenced by statistically significant improvements in exercise capacity and well-being, the intertrochanteric fracture rehabilitation program is beneficial. This case study represents a comprehensive rehabilitation program for people who have had post-fracture surgery.

## Introduction

Intertrochanteric fractures in adult patients are becoming more common as the population ages [1]. Osteoporosis, as well as falls, seem to be two significant risk factors for hip fractures in older adults. Infection, deep vein thrombosis, displacement, non-union, avascular necrosis, delusions, as well as post-traumatic arthritis are all possible complications after surgery [2]. The average lifespan of older adults has increased, which has resulted in a rise in such fractures. Additionally, plain radiography alone might not be sufficient to diagnose these fractures, necessitating a thorough physical assessment and radiographic interpretation [3].

According to reports [4], over half of the patients are still unable to come back to their pre-injury mobility condition. Physicians and physical therapists have indeed been worried about variables that impact functional recovery [4]. Even though there are a variety of surgical interventions for hip fractures, the key objectives are immediate rehabilitation and the resumption of pre-existing social duties. Initial stabilization of a hip fracture allows for immediate rehabilitation. It leads to better short-term treatment outcomes, such as the chance to return to independent living, a shorter hospital stay, and a lower likelihood of developing bedsores, as well as potentially decreased mortalities and postsurgical complications [5].

According to a survey, individuals with hip fractures who underwent isometric activities and specific physiotherapies appeared to recover physiologically quicker and also had a better quality of life [6]. In the continuum of care for patients with fragility fractures, physical therapy can be extremely important [7]. In the initial stages, the most significant physical therapy objectives for patients with orthopedic issues are pain management; decreasing edema and joint swelling; enhancing joint range of motion; tissue flexibility; prevention of muscle weakness and muscle atrophy; and in the later stages, muscle strengthening; improving balance; enhancing coordination, and developing motor control programs. Following fracture fusion, the patient must engage in long-term exercises to restore his or her muscular strength to the pre-fracture level [6]. This allows appropriate mobilization approaches and the prescription of organized exercise routines for maximizing functional capacity and lowering the risk of falls and additional fractures [7].

In this case study, we present a 52-year-old man who underwent open reduction and internal fixation post intertrochanteric fracture and required effective physical therapy following surgery to reduce the risk of complications. A comprehensive week-by-week rehabilitation approach is not provided in any prior study, and previous studies only offered a small number of physical therapy techniques for the management of such fractures. In response to this need, an integrated physiotherapy protocol with week-by-week treatment strategies was designed and is implicated in the management of intertrochanteric fracture. In addition, it will benefit other therapists for future guidance in providing such rehabilitation to help the patient return to their pre-injury state.

## Case presentation

Patient information

**Figure 1:**
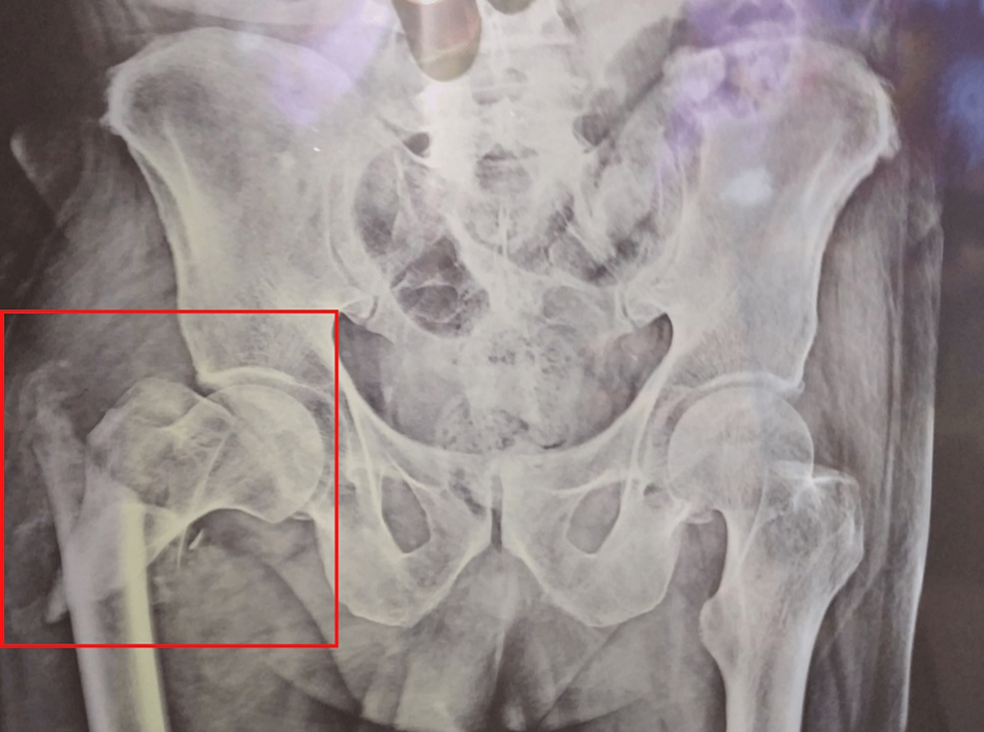
Display an X-ray showing an intertrochanteric fracture on the right side.

A 52-year-old man presented to the hospital with concerns about pain and swelling in his right hip since the morning. The patient reported an unexpected slip and fall while walking toward the washroom. The patient recalls a history of accompanying pain that began abruptly, progressed gradually, and was extremely painful, worsening with movement and improving with rest. The patient underwent an X-ray of the bilateral hip on the same day, which revealed an intertrochanteric fracture of the femur with subtrochanteric extension and no neuro deficit on the right side. Figure 1 displays an X-ray showing an intertrochanteric fracture on the right side.

A week before the fracture, the patient was diagnosed with COVID-19 and pneumonia, which was confirmed by several investigations. During this period, the patient had complaints of dyspnoea and cough along with early fatiguability and was bedridden for up to two weeks post COVID-19 diagnosis. Because he was in isolation, he received conservative treatment for his fracture, which included 4 kg of skin traction. After the period of isolation was over, the patient recovered well. Dyspnoea and cough complaints were resolved. Skin traction helped to relieve pain at the fracture site, allowing the patient to assume a semi-fowlers position with his head elevated to a 30-degree angle without bending his knees. Because of the sufficient recovery of the patient post-COVID-19, the patient was asked to be readmitted for further surgical management of the fracture. Until then, the fracture was managed conservatively with traction.

**Figure 2:**
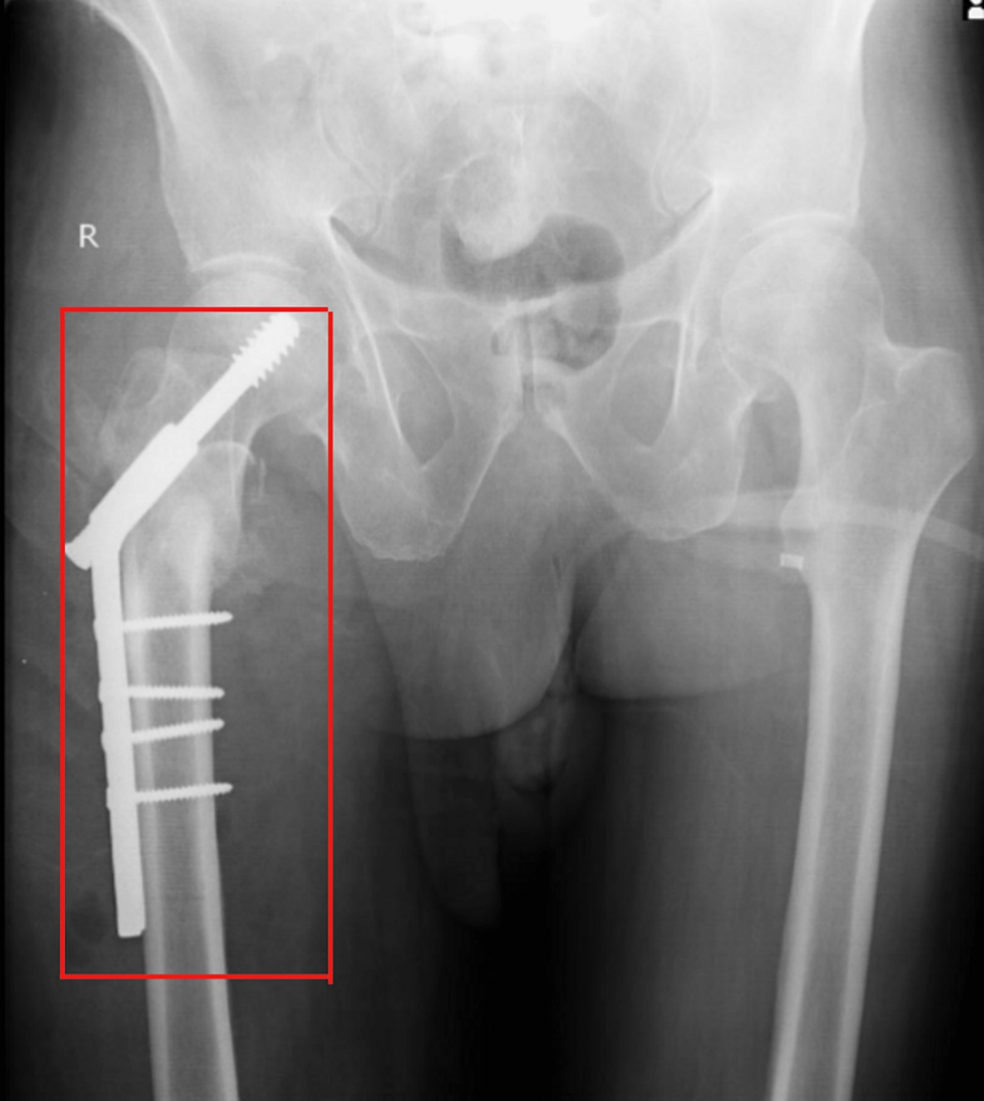
Display an anteroposterior view of an X-ray showing dynamic hip screw fixation of fracture fragments.

**Figure 3:**
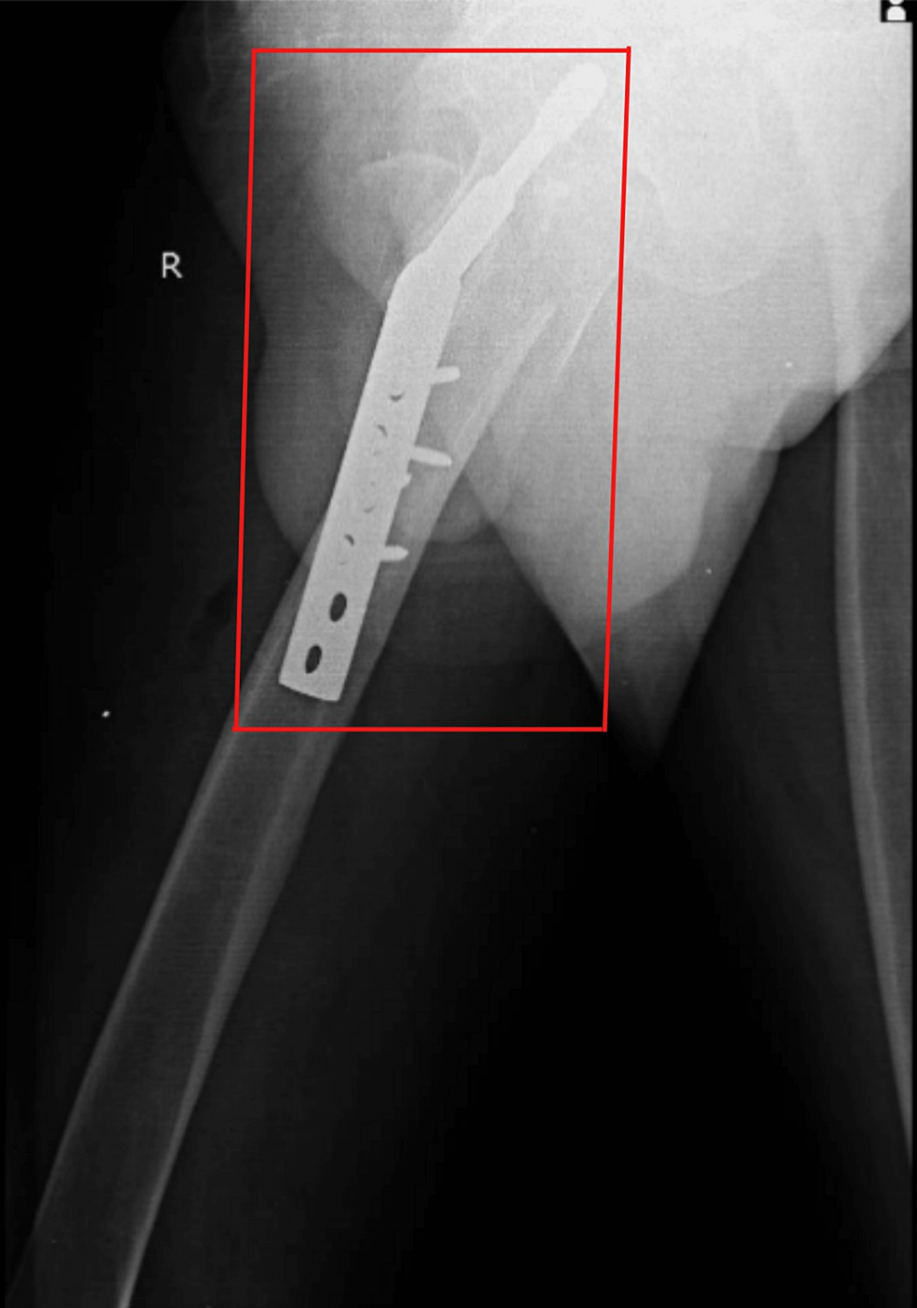
Displays a lateral view of an X-ray showing dynamic hip screw fixation of fracture fragments.

After one month, the patient was readmitted for further management, and the right-side femur fracture was treated with open reduction internal fixation (ORIF) and a dynamic hip screw (DHS), under spinal and epidural anesthesia. Figure 2 displays an anteroposterior view of an X-ray showing dynamic hip screw fixation of fracture fragments. Figure 3 displays a lateral view of an X-ray showing dynamic hip screw fixation of fracture fragments.

After two weeks of surgery, the patient was discharged as his vitals were stable, pain relief was present at the fracture site, the sutures had been removed, the suture site was healthy, active toe movements were present, and distal circulation was intact. The patient was also advised to come for follow-up after three weeks in the orthopedic outpatient department, and he continued his further physiotherapy treatment in the physiotherapy outpatient department.

Clinical findings

A physical evaluation was performed on postoperative day 3 with the patient's consent. He has been placed in a supine-lying position with his head elevated and his back properly supported. Both anterior superior iliac spines (ASIS) were at the same level. Throughout the inspection, the chest wall's mobility was noted to be diminished. The right lower limb, i.e., the affected leg, was slightly abducted and externally rotated with the knee in extension and the ankle in a neutral position. Diffuse swelling was present over the right hip joint.

On palpation, there was a rise in local temperature over the right hip along with grade 2 tenderness present over the greater trochanter. On further examination, a limb length discrepancy of 2 cm was noticed between the operated leg and the normal leg. Due to the presence of pain, hip and knee range of motion were not elicited. Active ankle-toe movements were present. Distal circulation was intact without any neuro deficit.

An 8 cm long horizontal incision on the lateral side of the right upper thigh, which was going to extend throughout the greater trochanter, was observed for dynamic hip screw fixation of the intertrochanteric fracture, and it was tender to the touch. The vastus lateralis was split after the deep fascia was cut. A dynamic hip screw of size 90 mm was inserted. A short-barrel 135-degree plate was fixed and the fracture was stabilized. The drain was inserted. Sutures were used to close the incision, and the wound was carefully dressed. The patient was having muscle weakness over the right thigh, early exhaustion on minor exercise, positional dependency, and mental anguish as a result of tenderness at the incision site and a lengthy hospital stay following hip surgery.

Physiotherapy interventions

The goal of an individual's rehabilitation would have been to allow him to come back to his normal daily activities with as little difficulty as possible. This patient received physiotherapeutic treatments for 12 weeks. Table 1 represents the physiotherapy management provided to the patient from week 1 to week 4. Table 2 represents the physiotherapy management provided to the patient from week 5 to week 8. Table 3 represents the physiotherapy management provided to the patient from week 9 to week 12. Figure 3 represents ambulation by the patient with the help of a walker.

**Table 1:** Summarizes physiotherapy management provided to the patient from week 1 to week 4. Reps: repetitions, BD: twice a day, TD: thrice a day, ROM: range of motion, AROM: active range of motion, SLR: straight leg raise, ADLs: activities of daily living.

Sr. No.	Physiotherapy treatment goals	Therapeutic intervention	Treatment regimen
1	To provide awareness of the condition, gain the cooperation and consent of the patient and his family members.	Patient and caregiver education and counseling about exercise regimen and the importance of adherence to it.	Positioning every 2 hours, early ambulation, and activity of daily living.
2	To prevent pulmonary, circulatory and integumentary complications post-surgery and to prevent limb rotation deformity.	(1) Manual positioning, half lying/semi-fowlers position was given initially; later upright sitting was given support with pillows -air beds	(1) Positioning after every 2 hours.
		(2) Ankle pumps	(2) Initially 25Reps × 1 set × BD. Later 25 Reps × 2 sets × TD.
3	To reduce pain at the fracture site	Cryotherapy	10 minutes each time 4-5 times daily.
4	To enhance the hip and knee joint’s ROM.	(1) AROM of Hip, knee in supine lying bilaterally.	Week 1–10 Reps × 1 set × TD. Week 2–15 Reps × 1 set × TD. Week 3 and 4 – 20 Reps × 1 set × TD.
5	To improve strength of muscles around hip and knee joint.	(1) Isometric exercises for quadriceps, glutei, and hamstrings.	Week 1 – 10 Reps × 1 set × TD. Week 2–15 Reps × 1 set × TD. Week 3 and 4 – 20 Reps × 1 set × TD.
		(2) Static exercises for quadriceps and hamstrings.	
		(3) Isotonic exercises for the ankle (gastrosoleus).	
		(4) SLR	
		(5) Dynamic quadriceps using TheraBand and weight cuff.	
6	To enhance the strength of the upper limb.	(1) Overhead arm flexion-extension with TheraBand.	Week 1 – 10 Reps × 1 set × TD. Week 2–15 Reps × 1 set × TD. Week 3 and 4 – 20 Reps × 1 set × TD.
		(2) Elbow curls with 1 kg weight-cuff.	
7	To initiate weight-bearing on the affected leg and ambulation.	(1) Pre-weight-bearing exercises - prone lying, four-point kneeling, knee walking.	(1) Week 1
		(2) Partial weight-bearing toe-touch weight-bearing.	(2) Week 2.
		(3) Ambulation with mobility aids such as a walker and stand pivot transfers.	(3) Week 2, 3, and 4.
8	To improve and modify the ADLs.	(1) Utilization of a lifted toilet seat as well as chair.	Follow from 2 to 4 week.
		(2) Trying to wear the pants on the injured leg first and then taking them off on the unaffected limb.	
		(3) Prior to getting out of bed, roll towards the unaffected side.	

**Table 2:** Summarizes physiotherapy management provided to the patient from week 5 to week 8.

Sr. No.	Physiotherapy treatment goals	Therapeutic intervention	Treatment regimen
1.	To improve and maintain functional ROM of hip and knee	(1) Self-assisted heel slides beyond 90°.	(1) Week 5–10 Reps × 1 set TD; week 6–15 Reps × 1 set TD; week 7 and 8–20 repetitions × 1 set TD.
		(2) Sit with the legs hanging over the edge of the bed.	(2) Initially sitting for 30 minutes, increasing time according to the patient's potential.
2.	To improve the endurance of muscles around the hip and knee joint	(1) Self-resisted exercises for hip and knee muscles.	Week 5–10 Reps × 1 set TD; week 6–15 repetitions × 1 set TD; week 7 and 8–20 repetitions × 1 set TD.
		(2) Isometric exercises for gluteus maximus and medius.	
		(3) Resisted exercises of hip flexion, extension, and abduction using TheraBand and weight cuff.	
3.	To improve the weight-bearing and gait of the patient	(1) Use of assistive devices during transfers and ambulation (Figure 4).	From the fifth week onwards, with the assistance of crutches, a three-point gait pattern was initiated.
		(2) Three-point gait pattern	
4.	To improve and modify the ADLs.	(1) Independent bed mobility.	From the fifth week, the patient begins to be self-sufficient.
		(2) Independent in dressing.	

**Table 3:** Summarizes physiotherapy management provided to the patient from week 9 to week 12. ROM: range of motion, TD: thrice a day, ADLs: activities of daily living.

Sr. No.	Physiotherapy treatment goals	Therapeutic intervention	Treatment regimen
1.	To maintain functional and regain regular hip and knee ROM.	(1) Hip and knee joints full active and passive ROM.	Multiple times in a day.
2.	To maintain the endurance of hip and knee muscles.	(1) Isotonic and isokinetic exercises to the hip and knee.	Weeks 9 and 10–20 repetitions × 1 set TD. Week 11 and 12–30 repetitions × 1 set TD.
		(2) Resisted hip flexion, extension, and abduction exercises using TheraBand and weight cuff.	
3.	To regain normal weight-bearing and gait pattern.	(1) Four-point gait pattern using crutches.	With the assistance of crutches, a four-point gait pattern was initiated, as well as independence in static balance on the affected limb, commencing in the ninth week.
		(2) Spot marching	
		(3) One-leg stand	
		(4) Full weight bearing on the affected limb.	
		(5) Stair climbing	
4.	To improve and modify the ADLs.	(1) Avoid limping in gait.	Precautions of this nature were followed by the patient.
		(2) Gait training with correct comfortable footwear and home practice in front of a postural mirror.	

**Figure 4:**
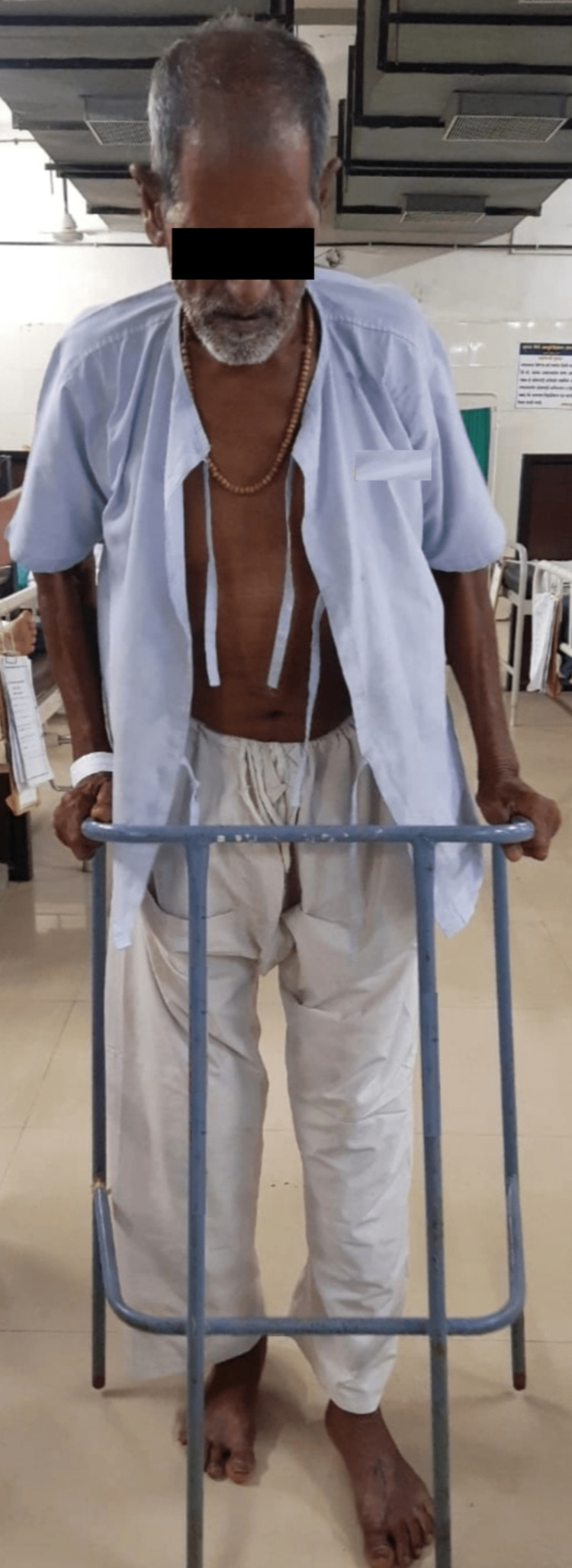
With the assistance of a walker, gait training was provided to the patient.

Intervention outcomes and follow-up

Pre-and post-rehabilitation outcome measures of response after 12 weeks of physiotherapy rehabilitation were taken. Table 4 shows the outcome measure scores.

**Table 4:** Pre and post-rehabilitation outcome measures. NPRS: numerical pain rating scale, ROM: range of motion, MMT: manual muscle testing.

Sr.No.	Outcome measures	Pre-physiotherapy rehabilitation score		Post-physiotherapy rehabilitation score	
1	NPRS	9		4	
2	Right hip ROM	Active	Passive	Active	Passive
	flexion	45°	50°	105°	110°
	extension	10°	15°	20°	25°
	abduction	0°–15°	0°–20°	0°–20°	0°–25°
	adduction	15°–0°	20°–0°	20°–0°	25°–0°
	internal rotation	15°	20°	20°	25°
	external rotation	10°	15°	20°	25°
3	Right knee ROM	Active	Passive	Active	Passive
	flexion	0°–110°	0°–115°	0°–115°	0°–120°
	extension	110°–0°	115°–0°	115°–0°	120°–0°
4	MMT for hip muscles	Grade (out of 5)		Grade (out of 5)	
	Flexors	2		4	
	Extensors	2		4	
	Abductors	2		4	
	Adductors	2		4	
5	MMT of knee muscles	Grade (out of 5)		Grade (out of 5)	
	Flexors	3		5	
	Extensors	3		5	
6	Harris hip score	70/100 (poor)		88/100 (good)	
7	Lower extremity functional scale score	20/80		60/80	

## Discussion

The patient, in this case, had an acute onset and steadily ongoing pain well into the right hip, which has been managed with ORIF and DHS. The rehabilitation objectives were formulated, beginning with gentle exercises and progressing to full weight-bearing ambulation through the use of a walker. Many of the exercises had been repeated thrice daily for ten repetitions, and ice packs had been applied to the patient's hips during the therapeutic process to relieve the exercise-induced discomfort. Though some studies have investigated the impact of post-hip surgery recovery on patients, there is a paucity of research on acute and routine treatments [8]. To prevent a re-occurrence of the fracture, the patient stopped sitting on the floor, avoided sitting cross-legged, and maintained both legs apart. As a result of these treatment regimens, the patient got benefits with better outcomes and well-being.

Regardless of the motivation, it is critical to begin muscle resistance training as quickly as possible within a week of surgery due to the muscle wasting reported by Suetta et al. in the first weeks [9]. According to one cohort study, increasing the intensity of acute inpatient physical therapy has been linked to a higher likelihood of trauma patients being discharged home, and it has also recently been found to be reliable, resulting in faster mobility advancements [10]​​​​​​.

Smith et al. [11] conducted a survey that found it to be safe and efficacious with the goal of early balancing and gait learning. Immediate action is often used to reduce the morbidity and mortality of aged people with hip fractures, as well as encourage functional rehabilitation. Restricting weight-bearing conditions post-surgery prolongs reliance on assistive devices as well as the individual's need to stay in an extended care facility, complicating the rehabilitation process. Pain relief, proper positional awareness, edema alleviation, bedsores prevention, and pneumonia prevention were all highlighted by Schmitt-Sody and Valle [12]. Gabriel discovered that physical rehabilitation has a massive influence on building confidence and advancing movement patterns in postoperative patients [13].

According to the current report, the patient received a very well-planned physical therapy rehabilitation program with multiple activities and resistive equipment from a competent musculoskeletal physiotherapist. Cryotherapy and pain relievers led to a considerable reduction in pain, allowing the patient to dedicate more time to rehabilitation, which was associated with subsequent improvements in joint movement and strength training, as well as clinical status. The physical therapy sessions were designed to preserve the right lower limb's muscle integrity while also enhancing the left lower limb as well as both upper limbs to encourage individual independent ambulation with an assistive device and very little aid for everyday routines. The patient was told to do most of the workouts as part of the home program, given a documented protocol, and told to come back for follow-up meetings.

The sole focus of this case report was to highlight the importance of quick surgical repair, as well as the necessity of physical therapy and rehabilitation services, and to offer an integrated protocol with week-by-week treatment strategies for the management of old age intertrochanteric fracture in order to meet the patient's performance goals and prognosis. No such study has been conducted previously, providing the complete week-wise physiotherapy rehabilitation programme for intertrochanteric fracture.

## Conclusions

The post-fracture rehabilitation program is effective, with substantial gains in physical functioning as well as well-being. The above case report provides a comprehensive strategy for the treatment of individuals who have had post-fracture surgery. The patient's full recovery had not been attained during the treatment period; however, the majority of the restorative aims have been encountered, including improved muscle strength, steadily increasing range of motion of the hip, increased functional capacity, pain reduction, improved gait pattern, and daily activities of the individual after 12 weeks of focused physical therapy treatment.
